# Exploring Acute Febrile Illness in Children: Clinical Characteristics and Diagnostic Challenges

**DOI:** 10.7759/cureus.58315

**Published:** 2024-04-15

**Authors:** Giridhar Darishetty, Vasudev Kompally, Vura U V Nagajyothi, Subhan B Bukkapatnam, Pratap Gudi, Arun Alugoya

**Affiliations:** 1 Department of Pediatrics, Kakatiya Medical College, Hanamkonda, IND

**Keywords:** diagnostic challenges, pediatrics, fever without foci, short duration, febrile illness

## Abstract

Background

Pediatric febrile illnesses are a major cause of hospital admissions and are often associated with significant morbidity and mortality. These illnesses pose a diagnostic challenge to both clinicians and laboratories. This study aims to explore the clinical characteristics of acute febrile illness in children and examine the effectiveness of various diagnostic techniques.

Methods

This prospective study was carried out at the Mahatma Gandhi Memorial Hospital, Warangal, India, from January 2020 to October 2022. It included children aged one month to 12 years.

Results

Out of 245 identified cases, 195 met the inclusion criteria. This study found that 18 patients (9.23%) suffered from serious bacterial infections (SBIs). In 63 patients (32.20%), the source of infection remained unidentified. Among those with SBI, UTIs were the most frequent. Bacteremia was identified in 2.5% of the patients.

Conclusion

SBIs were identified in 18 hospitalized children (9.23%), with UTIs being the most common SBI in children aged one to 36 months. Children in this age group presenting with toxic symptoms should be thoroughly evaluated for SBIs. The study also observed a higher prevalence of Gram-negative bacteremia compared to Gram-positive cases.

## Introduction

Fever, commonly defined as a body temperature exceeding 38.0°C (100.4°F), is a frequent clinical symptom, especially in children [1]. Low immunization rates, unaddressed comorbidities, and postponed medical interventions are some of the factors that make this problem worse in developing countries. Approximately 20% of pediatric consultations are attributed to febrile illnesses in infants and children [2]. While 12% of these cases are due to underlying infectious agents, fever’s differential diagnosis is broad, encompassing a variety of infectious and noninfectious causes [3]. Bacterial diseases, often associated with more severe outcomes, are a primary focus in diagnostic processes, although viral infections also contribute significantly to morbidity and mortality, especially in younger infants.

Pediatric fevers can be classified into three categories: fever from infection without a focus (absence of rash), fever with localized signs (absence of rash), and fever accompanied by a rash. Predominantly, these cases involve children under three years of age, a group highly susceptible to both minor and severe infectious diseases, ranging from viral respiratory illnesses to bacterial meningitis [4,5]. Despite detailed clinical histories and comprehensive examinations, identifying the source of infection can be challenging. Various criteria and protocols, such as the Boston Criteria, Philadelphia Protocol, Pittsburgh Guidelines, and Rochester Criteria, have been developed to assist clinicians in these situations [6]. Management strategies are then tailored based on clinical and laboratory assessments, categorizing patients into high- or low-risk groups and varying treatments from home observation to intravenous antibiotics.

Because viruses, bacteria, protozoa, rickettsia, and other noninfectious agents can cause acute febrile illness (AFI), diagnosing the cause of the fever can be challenging, especially in young children. This is especially true in places with few medical resources. Ambiguous symptoms and signs often lead to the irrational prescribing of antibiotics and antimalarials.

If not promptly diagnosed and treated, AFIs can be life-threatening. In under-resourced settings in developing and underdeveloped countries, this often results in preventable fatalities due to misdiagnosis.

Research on children with fever without source (FWS) in India is limited, with a lack of clarity on the prevalence of severe bacterial infections in these cases. Most existing FWS guidelines are derived from Western studies [7]. This study aims to identify the etiology of children presenting to a tertiary referral center without localizing symptoms, contributing vital data to this under-researched area.

## Materials and methods

A prospective study was carried out on children aged one month to 12 years admitted to the Mahatma Gandhi Memorial Hospital, a tertiary care center under Kakatiya Medical College, a government institute situated in Warangal, India, with an AFI of less than five days duration over 18 months from January 2020 to October 2022. Ethical clearance was obtained from the Institute Ethical Committee (approval number KMC/KIEC/Pediatrics/2020-005).

Children admitted with a fever of short duration (less than five days) with a temperature of more than 38°C and without any localizing signs were included in the study. Children with a history of antibiotic intake within 48 hours, children with any immunodeficiency state, children with chronic illnesses, children with fever 48 hours after admission, or parents not giving consent for the study were excluded from the study.

All the children who met the inclusion criteria and were included in the study provided detailed documentation of complaints, history, and treatment for current disease, prior history, socioeconomic status, and immunization status in a questionnaire. Nutritional status and vital signs were documented. A comprehensive clinical examination was performed. Positive and relevant negative results regarding general examination, cardiovascular systems, respiratory systems, gastrointestinal systems, and the central nervous system were reported.

All patients had their blood total count, differential count, hemoglobin, platelet count, peripheral smear, smear for malaria parasite, and urine microscopic examination for the presence of pus cells performed in our lab. A lumbar puncture was performed on children who had abnormal sensorium and convulsions. Dengue serology was submitted for suspected patients. Further testing, such as serology for enteric fever and leptospirosis, was performed on children who still had fever after seven days. An ultrasonography examination of the abdomen was performed on all individuals who had UTIs. The micturating cysto-urethrogram was completed as stated. All of the patients were followed up on until a definite diagnosis was made.

Statistical analysis

The statistical analysis mainly involved the calculation of percentages. Categorical variables have been expressed in percentages and continuous variables in the median and interquartile range. The chi-square test was used to find the association between categorical variables. The result was considered statistically significant when the p-value obtained was <0.05.

## Results

**Figure 1:**
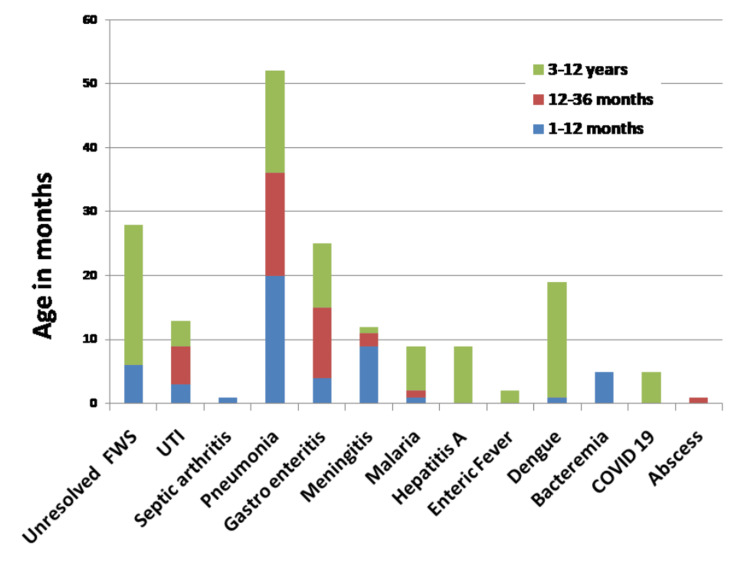
Proportions of age groups within each diagnosis FWS: fever without source

In this study, bacteremia was detected in 2.56% (n = 5) of patients, predominantly in the one- to three-month age group. Among these, five critically ill children were administered ceftriaxone as an empirical treatment, each exhibiting leukocyte counts above 15,000/microliter (Figure 1).

Blood culture sensitivity profiles indicated that *Klebsiella *isolates were mostly responsive to meropenem and amikacin but showed some resistance to cefotaxime and moderate resistance to ciprofloxacin in certain cases. *Staphylococcus aureus *showed sensitivity to cloxacillin, amikacin, and vancomycin. Citrobacter isolates were effectively treated with cefotaxime and amikacin. UTIs, often presenting as nonspecific fevers, were more frequently observed in female children. However, gender differences were negligible in those below three months. *Escherichia coli *were the most commonly isolated organism, particularly in uncircumcised male patients, one of whom had phimosis. Over half of the UTI patients had elevated white blood cell counts.

Urinary culture results revealed that *E. coli *have varied sensitivity to antibiotics, including ciprofloxacin, amikacin, and cefotaxime. *Klebsiella *showed mixed antibiotic responses, with some resistance noted. *Pseudomonas *was generally sensitive to ciprofloxacin and amikacin. Pneumonia, prevalent in the one- to 12- and one- to 36-month age groups, presented symptoms like fever, cough, feeding difficulties, and lethargy. Radiographic findings typically included patchy or homogenous opacities. Treatment with ampicillin and amikacin was largely effective.

**Figure 2:**
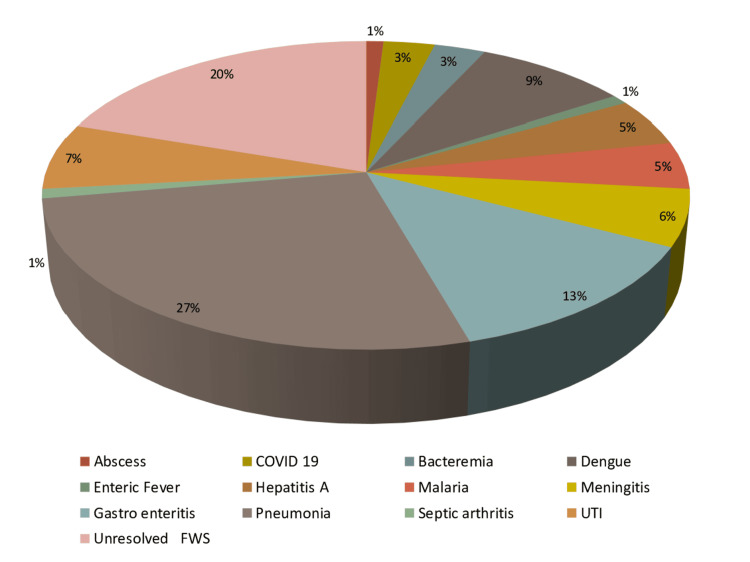
Distribution of total cases by diagnosis FWS: fever without source

Malaria, constituting 4.61% (n = 9) of cases, was mainly attributed to *Plasmodium vivax *and *Plasmodium falciparum*. Patients responded well to treatments such as artesunate and artemether-lumefantrine (Figure 2).

Children with meningitis underwent lumbar punctures, and diagnoses were confirmed through cerebrospinal fluid analysis. Treatment with ampicillin and ceftriaxone showed positive outcomes.

**Figure 3:**
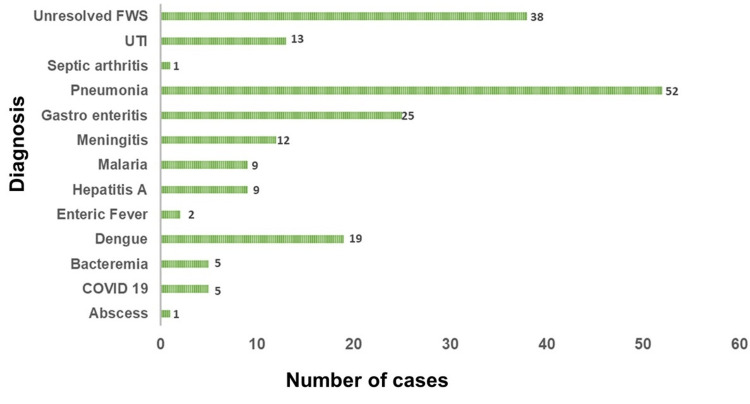
Total number of cases per diagnosis FWS: fever without source

Dengue fever, representing 9.74% (n = 19) of cases, varied in presentation from shock to petechial rash. Fluid management was the primary treatment approach. Hepatitis A, identified in 4.61% (n = 9) of cases, typically affects children over three years old, presenting with fever and jaundice. Treatment was conservative, and one severe case of hepatic encephalopathy recovered. FWS, found in 19.48% (n = 38) of cases, was presumed to be of viral origin due to its self-limiting nature. A subset of these cases exhibited gastroenteritis symptoms, mostly in children under 60 months (Figure 3).

Overall, the study revealed that the distribution of these illnesses varied with age, with pneumonia being the most common diagnosis across all age groups. Conditions like bacteremia and meningitis were notably more prevalent in the younger age groups (Table 1).

**Table 1:** Diagnosis cases among different age groups ^*^ p-value is calculated by considering age groups as categorical variables. The chi-square test was used to find the association between categorical variables. Fisher’s exact test is used for small sample sizes or when the assumptions of the chi-square test are not met. FWS: Fewer without source

	1–12 months		12–36 months		3-12 years			
Diagnosis	Number of cases	%	Number of cases	%	Number of cases	%	Total cases	%
Abscess	0	0.00%	1	0.51%	0	0.00%	1	0.51%
COVID-19	0	0%	0	0%	5	2.56%	5	2.50%
Bacteremia	5	2.50%	0	0%	0	0.00%	5	2.50%
Dengue	1	0.51%	0	0%	18	9.20%	19	9.20%
Enteric fever	0	0.00%	0	0%	2	1.02%	2	1.02%
Hepatitis A	0	0.00%	0	0%	9	4.60%	9	4.60%
Malaria	1	0.51%	1	0.51%	7	3.50%	9	4.60%
Meningitis	9	4.60%	2	1.02%	1	0.51%	12	6.10%
Gastroenteritis	4	2.05%	11	5.60%	10	5.12%	25	12.8%
Pneumonia	20	10.20%	16	8.20%	16	8.20%	52	26.60%
Septic arthritis	1	0.51%	0	0%	0	0.00%	1	0.51%
UTI	3	1.50%	6	3.07%	4	2.05%	13	6.60%
Unresolved FWS	6	3.07%	10	5.12%	22	11.20%	38	19.40%
TOTAL	50	25.60%	50	25.60%	95	48.70%	195	100%
p-value	0.0001*		0.0721*		0.0018*			

## Discussion

This study, conducted at the Mahatma Gandhi Memorial Hospital, a major tertiary center in northeast Telangana, focused on children aged one month to 12 years admitted with fever without localizing signs. Out of 245 cases initially identified, 195 children met the study’s inclusion criteria. The findings revealed that 18 patients (9.23%) had a serious bacterial infection (SBI), while in 63 cases (32.20%), the exact cause of the fever remained unidentified, suggesting a probable viral etiology other than dengue or COVID-19. UTI emerged as the most prevalent SBI, with bacteremia detected in 2.5% (n = 5) of patients.

The incidence of SBIs was relatively low at 9.23%, compared to higher rates reported in similar Western studies [8,9]. Comparisons with studies from various geographical regions, such as India [10] and Nigeria [11], which show varying prevalence and causative organisms for bacteremia and other infections, highlight this discrepancy.

UTIs were the most common bacterial infection observed across all age groups, with a higher incidence in females. International studies corroborate these findings, although the incidence rates vary. For instance, research from Brazil and the United States indicates varying prevalence rates of UTI in children with fever without localizing signs [12,13].

Additionally, the study found that bacterial meningitis was present in 6.1% (n = 12) of patients, with comparisons to international studies showing a range of prevalence and causative organisms [13,14]. Malaria was diagnosed in nine patients, with the prevalence increasing with age. This is consistent with other studies, although the prevalence in this study was lower than in some others [15,16]. A total of 19 patients (9.74%) had dengue fever diagnoses, which was most likely due to an epidemic that existed during the study period. This finding aligns with other studies that reported high incidences of dengue in similar clinical settings [17]. The study’s relatively small sample size limits the generalizability of the findings. Future research with a larger population could provide more conclusive results.

## Conclusions

This research at the Mahatma Gandhi Memorial Hospital explored the etiology of febrile illnesses in children aged one month to 12 years who exhibited no clear localizing symptoms. Key findings highlight a lower occurrence of SBIs (9.23%) compared to some Western studies, suggesting geographical variations. UTIs emerged as the most frequent SBI, particularly in females, which is consistent with international data. Bacterial meningitis was identified in 6.1% of the cohort, a rate that aligns with select global reports. Malaria’s prevalence corresponded with age increases, reflecting trends noted in other research, although it registered lower than some studies suggest. Dengue fever was diagnosed in 9.74% of the patients, likely linked to a simultaneous epidemic. Notably, a significant proportion (19.48%) of febrile episodes remained unexplained, were classified as FWS, and were typically self-resolving. Overall, the study offers valuable insights into pediatric fever causes in a resource-constrained setting in Northeast Telangana, revealing lower SBI rates compared to some Western counterparts, with UTIs and bacterial meningitis being the most common. It underscores the need for expanded research with more substantial cohorts and advanced diagnostics to reinforce these findings.
